# Microbial genome collection of aerobic granular sludge cultivated in sequencing batch reactors using different carbon source mixtures

**DOI:** 10.1128/mra.00102-24

**Published:** 2024-03-27

**Authors:** Jaspreet S. Saini, Aline Adler, Laëtitia Cardona, Pilar Natalia Rodilla Ramírez, Ruizhe Pei, Christof Holliger

**Affiliations:** 1 Laboratory for Environmental Biotechnology, Ecole Polytechnique Fédérale de Lausanne, Lausanne, Switzerland; Department of Biology, Queens College, Queens, New York, USA

**Keywords:** biofilms, aerobic granular sludge, metagenome-assembled genomes, wastewater, PacBio, accumulibacter, illumina

## Abstract

Aerobic granular sludge (AGS) consists of a microbial consortium that has an important role in wastewater treatment. This study investigates AGS microorganisms cultivated in a laboratory-scale sequencing batch reactor. Metagenomic sequencing was conducted using PacBio and Illumina, resulting in 759 metagenome-assembled genomes, 331 of which remained after dereplication.

## ANNOUNCEMENT

Aerobic granular sludge (AGS) is a promising wastewater treatment process for efficient biological carbon, nitrogen, and phosphorus removal (1). This study collected activated sludge from a wastewater treatment plant (ARA Thunersee, Switzerland) and cultivated it with different carbon source mixtures in a laboratory-scale sequential batch reactor (2).

Sample collection, DNA extraction, and sequencing protocols have previously been described in detail (3, 4). Briefly, biomass was collected on four different days and for each day, two DNA extractions were carried out, and used for Illumina (extraction A and B) and PacBio sequencing (extraction B) (Table 1). DNA “extraction (A)” was based on a customized CTAB protocol, while “extraction (B)” used the Maxwell 16 Tissue DNA Purification Kit (3, 4). Illumina samples were processed at the University of Lausanne, including library preparation and multiplex sequencing, using the HiSeq 2500 platform with paired-end mode and read lengths of 2 ×  100 bp (4). PacBio libraries, generated with the SMRTbell Template Prep Kit 1, underwent size-based selection using the Blue Pippin system (Sage Science, MA, USA). This involved choosing DNA molecules between 7 and 10 Kb size as per the methodology (4). Sequenced samples (Table 1) were processed using two workflows, referred to as “workflow A” and “workflow B” with default parameters of software unless specified otherwise.

**TABLE 1:** Twelve metagenomic samples with accessions, sequencing technology, DNA extraction, sampling day, carbon substrates, sequenced reads, and assembly statistics

Accession^*a*^	Sequencing technology^*b*^	DNA extraction^*c*^	Day^*d*^	Carbon susbstrates^*e*^	Number of reads^*f*^	Average read length^*g*^	Assemblies size (Mbp)^*h*^		Assemblies (N50)^*i*^		Assemblies (number of contigs)^*j*^	
ERR5621421	Illumina	A	71	Volatile fatty acids	61,579,556	101	260	258	28,719	29,682	19,427	19,361
ERR5621422	Illumina	B			51,182,028	101						
ERR5621427	PacBio	A			1,201,009	5,911	69		65,018		1,484	
ERR5621419	Illumina	A	322	Complex monomeric	60,911,008	101	260	257	18,714	19,478	24,455	24,064
ERR5621420	Illumina	B			49,723,068	101						
ERR5621428	PacBio	A			775,085	4,238	30		76,706		548	
ERR5621423	Illumina	A	427	Complex monomeric	56,540,510	101	360	358	17,470	17,265	32,953	33,060
ERR5621424	Illumina	B			49,921,504	101						
ERR5621429	PacBio	A			1,329,387	5,286	76		55,509		1,997	
ERR5621425	Illumina	A	740	Complex polymeric	62,194,586	101	497	493	24,172	23,187	37,625	38,376
ERR5621426	Illumina	B			57,070,578	101						
ERR5621430	PacBio	A			1,149,988	7,878	124		211,407		1,299	

^*a*^ Accession numbers with download links associated with BioProject accession number PRJEB38840.

^*b*^ Type of metagenomic sequencing technology: Illumina short reads and PacBio long reads.

^*c*^ DNA extraction method.

^*d*^ Day of sampling.

^*e*^ Type of carbon source mixtures.

^*f*^ Number of Illumina and PacBio reads calculated using the SeqKit.

^*g*^ Lengths of Illumina and PacBio reads calculated using the SeqKit.

^*h*^ Size of metagenomic assemblies were calculated using BBMap. The minimum contig length was 2,500 bp.

^*i*^ N50 values of the metagenomic assemblies were calculated using BBMap. The minimum contig length was 2,500 bp.

^*j*^ The number of contigs in the metagenomic assemblies was determined using BBMap with a minimum contig length of 2,500 bp.

Assembly and binning methods for “workflow A” have previously been published (3, 4), which reports six high-quality Accumulibacter-related MAGs, indicating high microdiversity within this genus (4). In “workflow A”, Illumina short reads were trimmed and filtered for quality using Trimmomatic (v 0.36) (5), whereas “workflow B” employed Atlas (v 2.22) (6) and LongQC (v 1.2.0) (7) for adapter, quality trimming, and results visualization. In "workflow B", SPAdes (8) (v 3.15.5, -pacbio and -meta) obtained hybrid assemblies by incorporating all three samples for each day—one PacBio and two Illumina data sets. Binning was employed using CONCOCT (v 1.1.0) (9), MetaBAT 2 (v 2.15) (10), and MaxBin 2 (v 2.2.7) (11) with default settings in Anvi’o (v 7.1) (12), using coverage information from all 12 sequencing samples (Table 1). SeqKit (2.4.0) (13) and BBmap (v 39.01) (14) calculated the statistics on reads, assemblies, and MAGs, where N50 and L50 represent the length and count of the shortest contigs covering at least 50% of the sequences (15).

**Fig 1:**
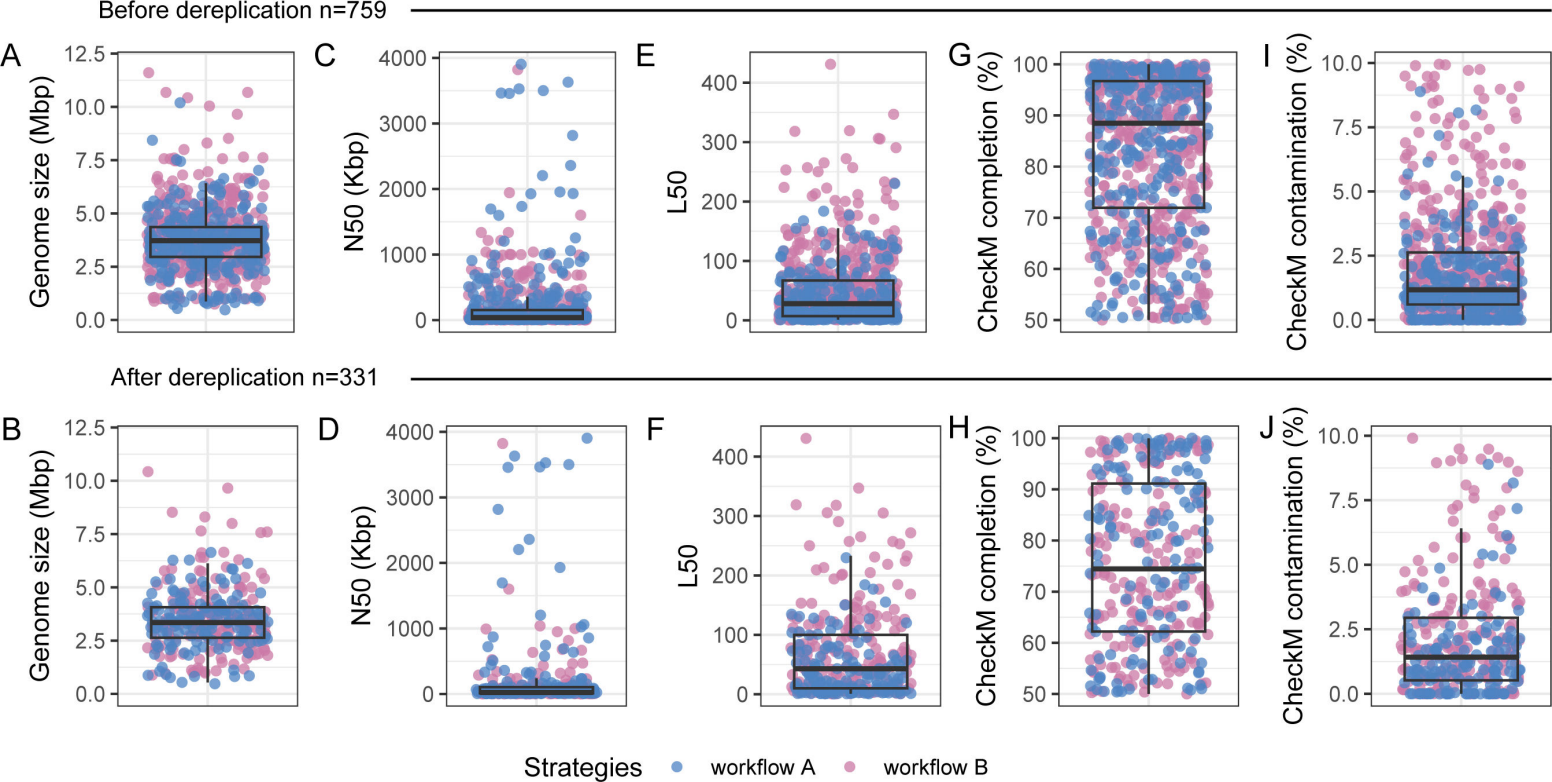
A summary of the MAGs collection before (*n* = 759) and after dereplication (*n* = 331) as described in two panels. It includes BBMap-based statistics and CheckM-based quality assessment. The statistics comprise (**A-B**) genome size and (**C-F**) N50 and L50 values. (**G-J**) Assessment of MAGs completion and contamination by CheckM. The colors indicate the different strategies described as workflows in methods.

The two workflows together yielded 759 quality-controlled MAGs after using >50% completion and <10% contamination criteria in CheckM (v 1.12.1) (16). After post-dereplication using dRep (v 3.4.0, -pa 0.99, -ignoreGenomeQuality) (17), 331 MAGs remained. Before and after dereplication (Fig. 1A through J), the data sets had a median genome size, N50 value, L50 value, completion, and contamination of 3.72 and 3.35 Mbp (Size), 39.57 and 23.45 Kbp (N50), and 28 and 43 (L50), 88.47 and 74.45% (completion), and 1.17, 1.42% (contamination), respectively. Accumulibacter (*n* = 19), Nitrosomonas (*n* = 12), Propionivibrio (*n* = 11), Daejeonella (*n* = 9), and Azonexus (*n* = 8) were frequently occurring genera based on GTDB (Toolkit v 2.1.1, RS207) post-dereplication (18). The entire genome collection is now available for future studies investigating AGS metabolism.

## Data Availability

The National Center for Biotechnology Information (NCBI) database provides access to raw DNA sequences under the project PRJEB38840. These sequences are identified by accession numbers ranging from ERR5621419 to ERR5621430 as listed in Table 1. In addition, the metagenomic assemblies and MAGs collection with metadata, along with the R-script (R-studio v 2023.03.1+446) used for generating the figure, were deposited at Zenodo (10.5281/zenodo.10229272) (19).
